# β-adrenergic receptor inhibits heart regeneration by downregulating *Yap* m6A modification

**DOI:** 10.1038/s41419-025-07642-9

**Published:** 2025-04-14

**Authors:** Kaihang Guan, Zijian Li

**Affiliations:** 1 Department of Cardiology and Institute of Vascular Medicine, Peking University Third Hospital, Beijing Key Laboratory of Cardiovascular Receptors Research, Key Laboratory of Cardiovascular Molecular Biology and Regulatory Peptides, Ministry of Health, State Key Laboratory of Vascular Homeostasis and Remodeling, Peking University, Research Unit of Medical Science Research Management/Basic and Clinical Research of Metabolic Cardiovascular Diseases, Chinese Academy of Medical Sciences, Beijing, China; 2 Department of Pharmacy, Peking University Third Hospital, Beijing, China

**Keywords:** Cardiovascular diseases, RNA

## Abstract

Newborn mammals transiently maintain the heart regenerative capacity. β-adrenergic receptor (β-AR) is the most critical receptor in regulating cardiomyocyte behavior. However, the role and mechanism of β-AR, especially the subtypes of β-AR, in heart regeneration remain unclear. Here, we reveal that β-AR inhibits heart regeneration by downregulating *Yap* m6A modification. The β-AR expression is associated with heart regenerative capacity. After apical resection, β-AR (including β_1_-AR and β_2_-AR) inhibits heart regeneration. β_2_-AR exerts a more potent inhibitory effect compared with β_1_-AR. Mechanistically, both β_1_-AR and β_2_-AR downregulate *Yap* m6A modification and then YAP expression differentially by reducing METTL14 and IGF2BP1, respectively. Elevation of *Yap* m6A modification with adenoviruses encoding METTL14 and IGF2BP1 rescues YAP expression and cardiomyocyte proliferation inhibited by β_1_-AR and β_2_-AR, respectively. These findings indicate that both β_1_-AR and β_2_-AR inhibit heart regeneration in a m6A-dependent manner and reveal subtype-specific mechanism. These results will provide a new intervention strategy for heart regeneration.

## Introduction

Heart disease remains the leading cause of death worldwide [1]. After cardiac injury, the adult heart loses the functional myocardium due to the extremely limited regenerative capacity, resulting in an irreversible decline in cardiac function. Unlike adult mammals, newborn mammals can completely regenerate the heart through robust cardiomyocyte proliferation [2–4]. Revealing the mechanism of heart regeneration in neonatal mammals will provide potential therapeutic strategy for adult cardiac injury.

The sympathoadrenal system is the most important system to regulate cardiac activities [5]. β-adrenergic receptor (β-AR), as the most critical gateway to the sympathoadrenal system, mediates extremely critical pathophysiological signaling of cardiomyocytes [6], and β-blockers have become the basic drug for heart disease [7, 8]. Sympathetic hyperactivation is often triggered after cardiac injury [9, 10]. It is widely accepted that the use of β-blockers can improve cardiac function after cardiac injury [8]. Moreover, β_1_-AR inhibition could enhance heart regeneration post myocardial infarction in juvenile mice [11]. However, the role and mechanism of β-AR, especially the subtypes of β-AR, in heart regeneration remain unclear.

The Hippo pathway is highly conserved to control cell proliferation [12]. As the central effector of Hippo pathway, YAP is widely recognized as one of the most critical factors in promoting heart regeneration [13–15]. In recent years, *YAP* mRNA has been found to be regulated by m6A modification. *YAP* m6A modification mediated by m6A writers METTL3/METTL14, eraser ALKBH5, and readers IGF2BP1/IGF2BP2/YTHDF1 is involved in osteogenesis, cancers, and ischemic reperfusion injury [16–20]. However, the role of *YAP* m6A modification in heart regeneration remains to be elucidated.

In this study, we showed that increased expression of β-AR is associated with the loss of heart regenerative capacity. Activation of β-AR, including β_1_-AR and β_2_-AR, inhibits heart regeneration after apical resection. β_2_-AR exerts a more potent inhibitory effect compared with β_1_-AR. Mechanistically, both β_1_-AR and β_2_-AR reduce YAP expression to different levels by downregulating the m6A modification and stability of *Yap* mRNA differentially. The downregulated *Yap* m6A modification results from the decreased expression of METTL14 and IGF2BP1induced by β_1_-AR and β_2_-AR, respectively. Overexpression of METTL14 and IGF2BP1 could rescue *Yap* m6A modification, YAP expression, and cardiomyocyte proliferation inhibited by β_1_-AR and β_2_-AR, respectively. These findings indicate that both β_1_-AR and β_2_-AR inhibit heart regeneration in a m6A-dependent manner and reveal subtype-specific mechanism. These results will provide a new intervention strategy for heart regeneration.

## Materials and methods

### Transcriptomic data retrieval and analysis

To explore the correlation between the expression of β-AR and heart regenerative capacity, we retrieved and downloaded transcriptomic data (GSE107760) from the National Center for Biotechnology Information Gene Expression Omnibus (GEO) database (https://www.ncbi.nlm.nih. gov/geo/). The FPKM values of cardiac *Adrb1* and *Adrb2* in P1 and P7 mice were specifically screened. Z-score was calculated for each gene between the P1 and P7 mice. Student’s *t*-test was used to determine significance.

### Apical resection and drug treatment

All animal studies were approved by the Peking University Third Clinical Medical School Ethical Committee of Animals (LA2024088). All procedures conform to the guidelines from the National Institutes of Health Guide for the Care and Use of Laboratory Animals. The C57BL/6J mice used for study were purchased from Vital River Laboratory Animal Technology Co., Ltd (Beijing, China).

Postnatal day 1 (P1) mice of both sexes were subjected to apical resection. In general, P1 mice were anesthetized on an ice bed for 2–3 min. A transverse skin incision was performed in the left parasternal fourth intercostal space, followed by blunt dissection of the intercostal muscles in the third or fourth intercostal space to expose the intrathoracic cavity. A steady pressure was applied to the abdomen, thereby pushing the heart out of the chest. The cardiac apex was resected using iridectomy scissors to make the left ventricle chamber just exposed. The thoracic wall and skin incision were closed immediately using 8–0 non-absorbable sutures. The operated mice were then placed on a blanket at 37 °C for several minutes until resuscitation. For drug treatment after apical resection, saline or the following drugs were administered subcutaneously according to the specific designs: isoproterenol (ISO, HY-B0468, MCE, New Jersey, USA), propranolol (Prop, HY-B0573, MCE), dobutamine (DOB, HY-15746, MCE), vilanterol (VI, HY-14300A, MCE), metoprolol (MET, HY-17503, MCE), and ICI-118551 (ICI, HY-13951, MCE), all at a dose of 10 mg/kg/day.

### Echocardiography analysis

Echocardiography analysis was performed before hearts were harvested at 21 days post-resection (21 dpr). Mice were anesthetized with isoflurane (Baxter International Inc., Illinois, USA). Images were captured by a VisualSonics high-resolution Vevo 2100 system (VisualSonics Inc., Toronto, Canada). Two-dimensional parasternal short-axis views representing the minimum LV length were obtained in B-mode, and then the M-mode images reflecting the cardiac systolic function were captured. The fractional shortening (FS) was automatically calculated using the Visual Sonics Vevo imaging software.

### Histological analysis

Mice were sacrificed at 21 dpr, and hearts were harvested. Hearts were fixed in 4% paraformaldehyde overnight, dehydrated, and then embedded in paraffin. In the coronal plane, the heart was cut into consecutive sections with a thickness of 5 μm until the scar was exposed. Sections were further applied for Masson’s staining according to standard procedures.

### Isolation and culture of neonatal mouse cardiomyocytes (NMCMs)

Primary NMCMs were isolated from neonatal C57BL/6 J mice. Specifically, the hearts of P1 mice were harvested and then NMCMs were isolated using the Neonatal Heart Dissociation Kit (Miltenyi Biotec, 130-098-373, Bergisch Gladbach, Germany). After red blood cell lysis and filtration, cells were plated into 10 cm culture dishes for 2 h to allow cardiac fibroblast adherence. The suspended NMCMs were collected and cultured in Dulbecco’s modified essential (DMEM) medium supplemented with 15% fetal bovine serum and 1% penicillin-streptomycin in a humidified incubator at 37 °C in 95% air/5% CO_2_ for further experiments.

### Drug treatment and adenovirus transfection of NMCMs

After 24 h of culture, NMCMs were treated with saline or the following drugs for 48 h according to the specific designs: ISO, Prop, DOB, and VI, all at a concentration of 10 μM. For adenovirus transfection, adenovirus vectors encoding METTL14 or IGF2BP1 were transfected into NMCMs for 24 h before drug treatment. Adenoviral vectors encoding METTL14 or IGF2BP1 were constructed from Hanheng Biotechnology (Shanghai) Co., Ltd (Shanghai, China).

### Immunofluorescence staining

For hearts harvested at 7 days post-resection (7 dpr), 4% paraformaldehyde fixation overnight, dehydration, and OCT embedding were performed, and the hearts were then cut into slices with a thickness of 8μm. For cultured NMCMs, cells were fixed with 4% paraformaldehyde for 15 min at room temperature. Next, the slices or cells were permeabilized and blocked with PBS containing 0.3% Triton X-100 and 5% BSA for 1 h at room temperature, followed by incubation with primary antibodies at 4 °C overnight. The following primary antibodies were used: α-actinin (ab9465, Abcam, Cambridge, UK), Ki67 (ab16667, Abcam), phospho-histone H3 (06-570, Merck Millipore, Darmstadt, Germany), Aurora B (ab2254, Abcam). After being washed for 3 times with PBS and incubated with fluorescent secondary antibodies, the slides and cells were mounted with medium containing DAPI. Images were captured by Zeiss LSM 780 confocal laser scanning microscope (Carl Zeiss, Oberkochen, Germany).

### Western blot

NMCMs were lysed in RIPA lysis buffer containing protease inhibitors. Total protein (20 μg) was fractionated by SDS–PAGE, and then transferred to a nitrocellulose membrane. Membranes were blocked in 5% non-fat dry milk for 1 h. After washing three times with TBST, target membrane bands were incubated with primary antibodies at 4 °C overnight. The following primary antibodies were used: YAP (66900-1-Ig, Proteintech, Wuhan, China), METTL14 (ab309096, Abcam), IGF2BP1 (ab290736, Abcam), GAPDH (5174, Cell Signaling Technology, Massachusetts, USA). After washing three times with TBST, membrane bands were incubated with horseradish peroxidase-linked secondary antibodies. Finally, blots were detected using an ECL kit (WBKLS0500, Merck Millipore). Densitometry analyses were done with ImageJ software (National Institutes of Health, Bethesda, MD).

### Quantitative real-time polymerase chain reaction (qPCR)

Total RNA was isolated from cultured NMCMs using RNA isolater Total RNA Extraction Reagent (R401-01, Vazyme, Nanjing, China), and then 1 µg RNA was reverse-transcribed into cDNA using the HiScript III RT SuperMix for qPCR (R323-01, Vazyme). Then qPCR was conducted using Taq Pro Universal SYBR qPCR Master Mix (Q712-03, Vazyme). Relative mRNA levels were normalized to GAPDH. The sequences of the primers used for qPCR are in Table S1.

### Methylated RNA immunoprecipitation (MeRIP)-qPCR

EpiMark^®^ N6-Methyladenosine Enrichment Kit (E1610, NEB, Massachusetts, USA) was applied for the MeRIP assay. Briefly, total RNA was isolated from pretreated NMCMs and was fragmented by ultrasound. One-tenth of the total RNA was saved as the input control. The rest of the RNA was then immunoprecipitated with magnetic beads pre-coated with anti-m6A antibody (E1610, NEB) or anti-rabbit IgG (2729, Cell Signaling Technology). Then Monarch^®^ RNA Cleanup Kit (T2030, NEB) was applied to elute the m6A-modified RNA fragments. Further m6A enrichment was evaluated by qPCR, and the corresponding m6A enrichment was normalized to the input. Specific primers of *Yap* were designed as per the SRAMP website (http://www.cuilab.cn/sramp) analysis:

F, 5′-CAAAATGTCAGGAATTAGCT-3′;

R, 5′-ATAGGTGCCACTGTTAAGAA-3′.

### RNA stability assay

Isolated NMCMs were cultured and treated with drugs as above. After treated with 5 μg/ml actinomycin D (15021, Cell Signaling Technology) to block transcriptions, cells were collected at 0 h, 4 h, and 8 h post-treated, and total RNAs were isolated. Then qPCR was used to measure the relative mRNA levels at different timepoints.

### RNA immunoprecipitation (RIP)

Magna RIP Kit (17-700, Merck Millipore) was applied for the RIP assay. Briefly, NMCMs were washed and lysed in RIP lysis. After centrifugation, one-tenth of the total lysate was saved as the input control. The rest of the lysate was immunoprecipitated with magnetic beads pre-coated with anti-METTL14/IGF2BP1 antibody or anti-rabbit IgG at 4 °C overnight. Then magnetic beads were washed by RIP wash buffer, followed by proteinase K buffer treatment. Immunoprecipitated RNA was finally purified and evaluated by qPCR. The immunoprecipitated RNA was normalized to the corresponding input control.

### Statistical analysis

Data were processed and analyzed using GraphPad Prism version 8.0. The values were presented as the means ± SEM. Differences between two groups were analyzed by the two-tailed Student’s *t*-test. Differences between three or more groups of single-variable experiments were analyzed by one-way ANOVA with Tukey’s post-hoc test. Differences between groups in the bivariate experiment were analyzed using two-way ANOVA with Tukey’s post-hoc test. A value of *P* < 0.05 was considered statistically significant.

## Results

### The expression of β-AR is associated with heart regenerative capacity

**Fig. 1:**
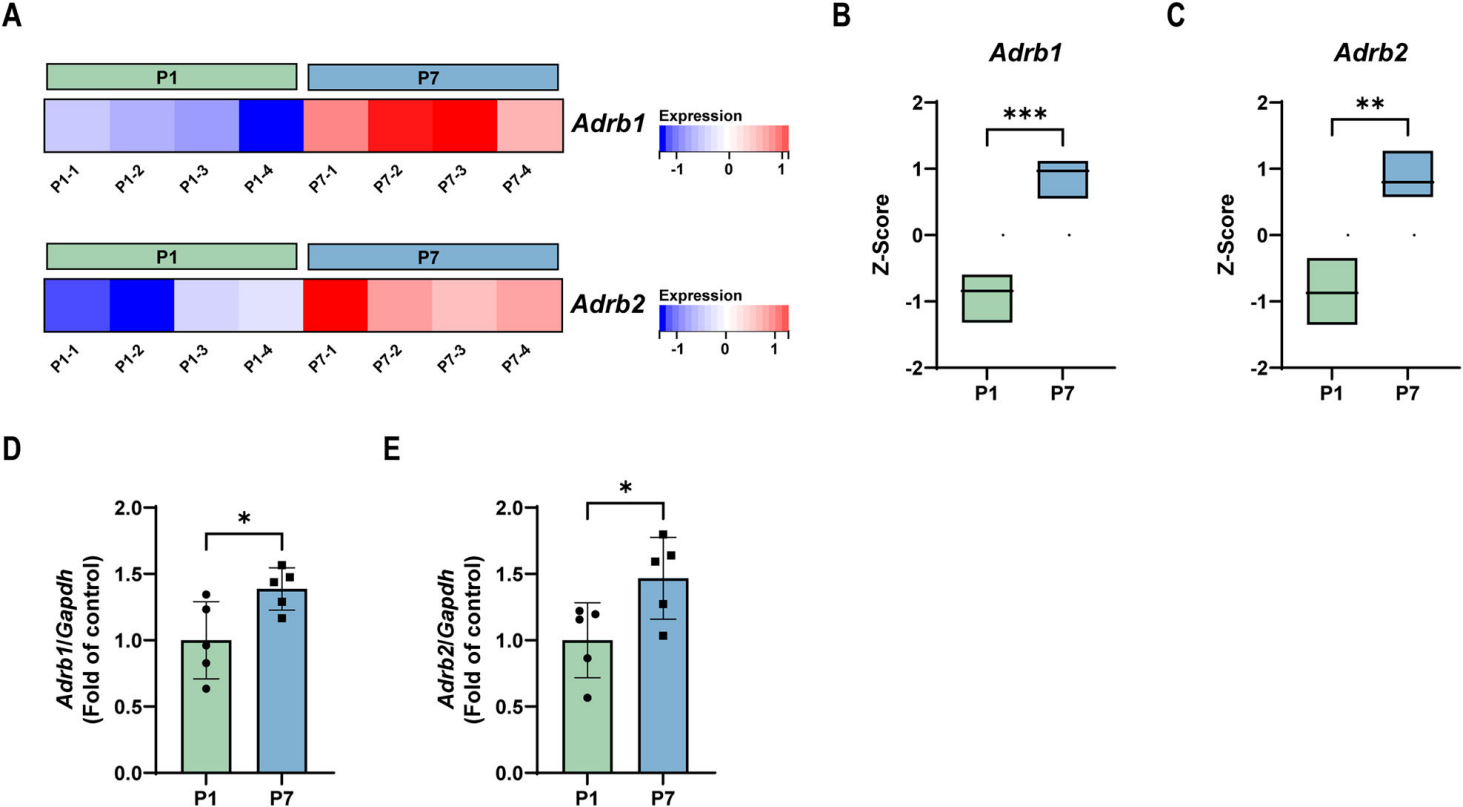
The expression of both β_1_-AR and β_2_-AR is increased at P7 compared with P1. **A** Transcriptomic heat maps of cardiac *Adrb1* (β_1_-AR) and *Adrb2* (β_2_-AR) at P1 and P7 (*N* **=** 4). FPKM data were analyzed using the public transcriptomic database from GEO datasets (GSE107760). Box plots of cardiac *Adrb1* (**B**) and *Adrb2* (**C**) at P1 and P7 according to the transcriptomic data. Box plots indicate the standardized numerical range in the group with the central line representing the median. QPCR analysis of β_1_-AR (**D**) and β_2_-AR (**E**) mRNA levels in heart at P1 and P7 (*N* **=** 5). Quantitative results were shown as mean ± SEM. Statistical analyses were performed by Student’s *t-*test. *P **<** 0.05, **P **<** 0.01, ***P **<** 0.001.

Mice generally lose the ability of heart regeneration at postnatal day 7 (P7) [4]. To explore the potential effect of β-AR on heart regeneration, we first analyzed the correlation between the expression of β-AR and heart regenerative capacity using the public transcriptomic data from GEO datasets (GSE107760) [21]. Results showed that compared with P1, the expression of both β_1_-AR and β_2_-AR in heart was markedly increased at P7 (Fig. 1A–C). Furthermore, qPCR confirmed that mRNA levels of both β_1_-AR and β_2_-AR in heart were increased at P7 compared with P1 (Fig. 1D, E). These results suggested that both β_1_-AR and β_2_-AR are involved in heart regenerative capacity.

### β-AR inhibits heart regeneration

**Fig. 2:**
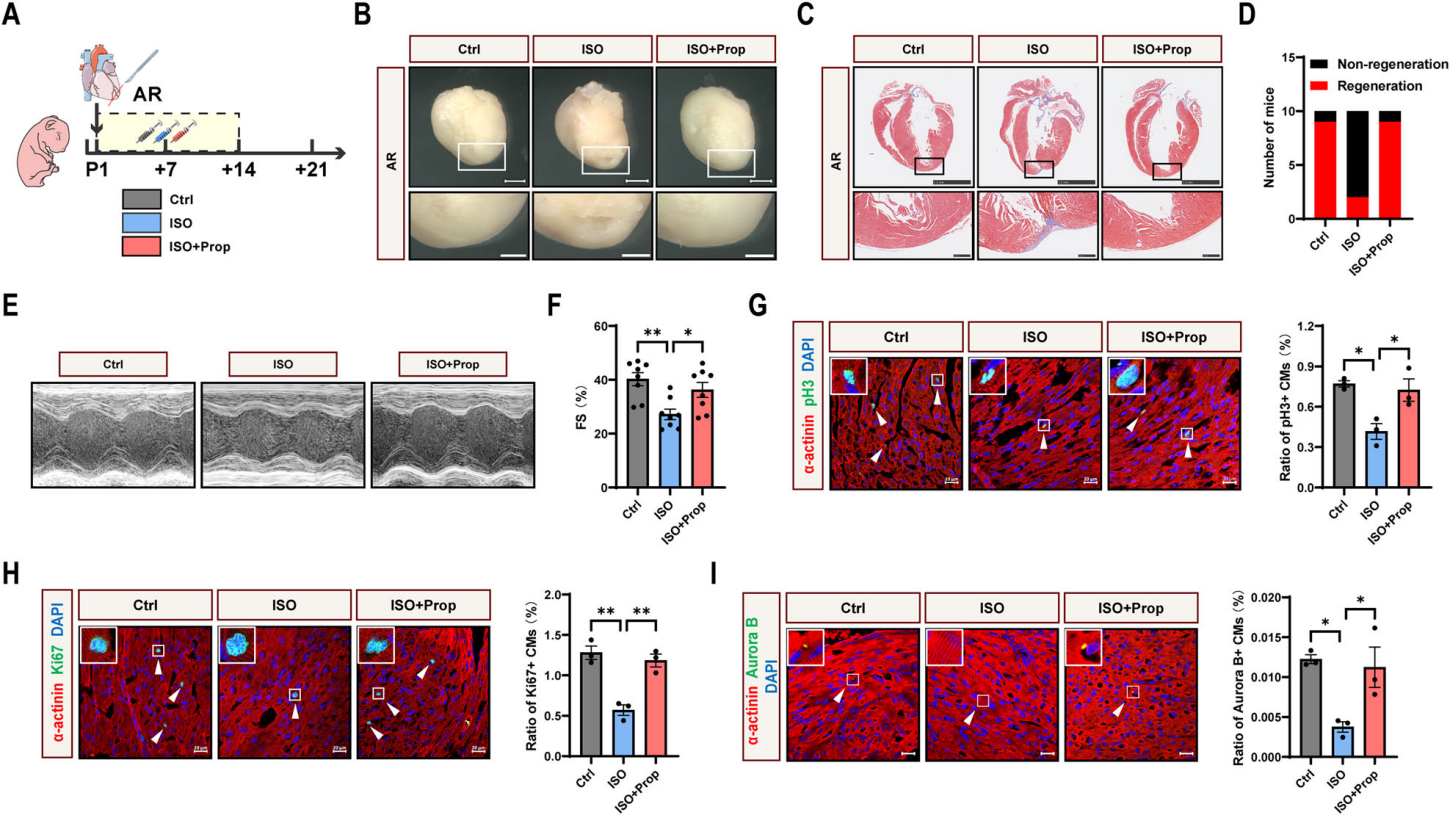
β-AR inhibits heart regeneration after apical resection. **A** Schematic representation of apical resection (AR) and drug injection for P1 mice. Ctrl, control; ISO, isoproterenol, β-AR agonist, 10 mg/kg/d; Prop, propranolol, β-AR antagonist, 10 mg/kg/d. **B** Gross morphology of hearts harvested at 21 dpr. Scale bars: upper, 200 μm; below, 100 μm. **C** Masson staining of hearts harvested at 21 dpr. Scale bars: upper, 2.5 mm; below, 250 μm. **D** Statistics of the number of regenerated hearts at 21 dpr (*N* = 10). **E**, **F** Echocardiography analysis of fractional shortening (FS) at 21 dpr (*N* = 8). Immunofluorescence staining of the mitosis marker pH3 (**G**), Ki67 (**H**), and Aurora B (**I**) (green), accompanied with cardiac α-actinin (red) and DAPI (blue) at 7 dpr (*N* = 3). Arrows show cardiomyocytes positive for pH3, Ki67, and Aurora B. CM, cardiomyocyte. Scale bars, 20 μm. Quantitative results were shown as mean ± SEM. Statistical analyses were performed by one-way ANOVA with Tukey’s post-hoc test. *P < 0.05, **P < 0.01.

To investigate whether β-AR is essential for heart regeneration, P1 mice were subjected to apical resection, followed by injection of isoproterenol (ISO, β-AR agonist) and propranolol (Prop, β-AR antagonist) (Fig. 2A). Results showed that ISO significantly impaired heart regenerative capacity at 21 days post-resection (dpr), and Prop counteracted the suppressive effect induced by ISO (Fig. 2B–D, and S1A). Moreover, the inhibitory effect of ISO on heart regeneration was in a dose-dependent manner (Fig. S2). Echocardiography analysis revealed that ISO impeded recovery of systolic function at 21 dpr, and the effect was reversed by Prop (Fig. 2E, F). We further examined cardiomyocyte proliferation at 7 dpr, the time point of peak cardiomyocyte proliferation after cardiac injury [4]. Immunofluorescence staining showed that ISO reduced the proliferation markers (pH3, Ki67, and Aurora B) in heart tissue, and the effect was also reversed by Prop (Fig. 2G–I). Taken together, these results indicated that β-AR inhibits heart regeneration after apical resection.

### Both β_1_-AR and β_2_-AR inhibit heart regeneration

**Fig. 3:**
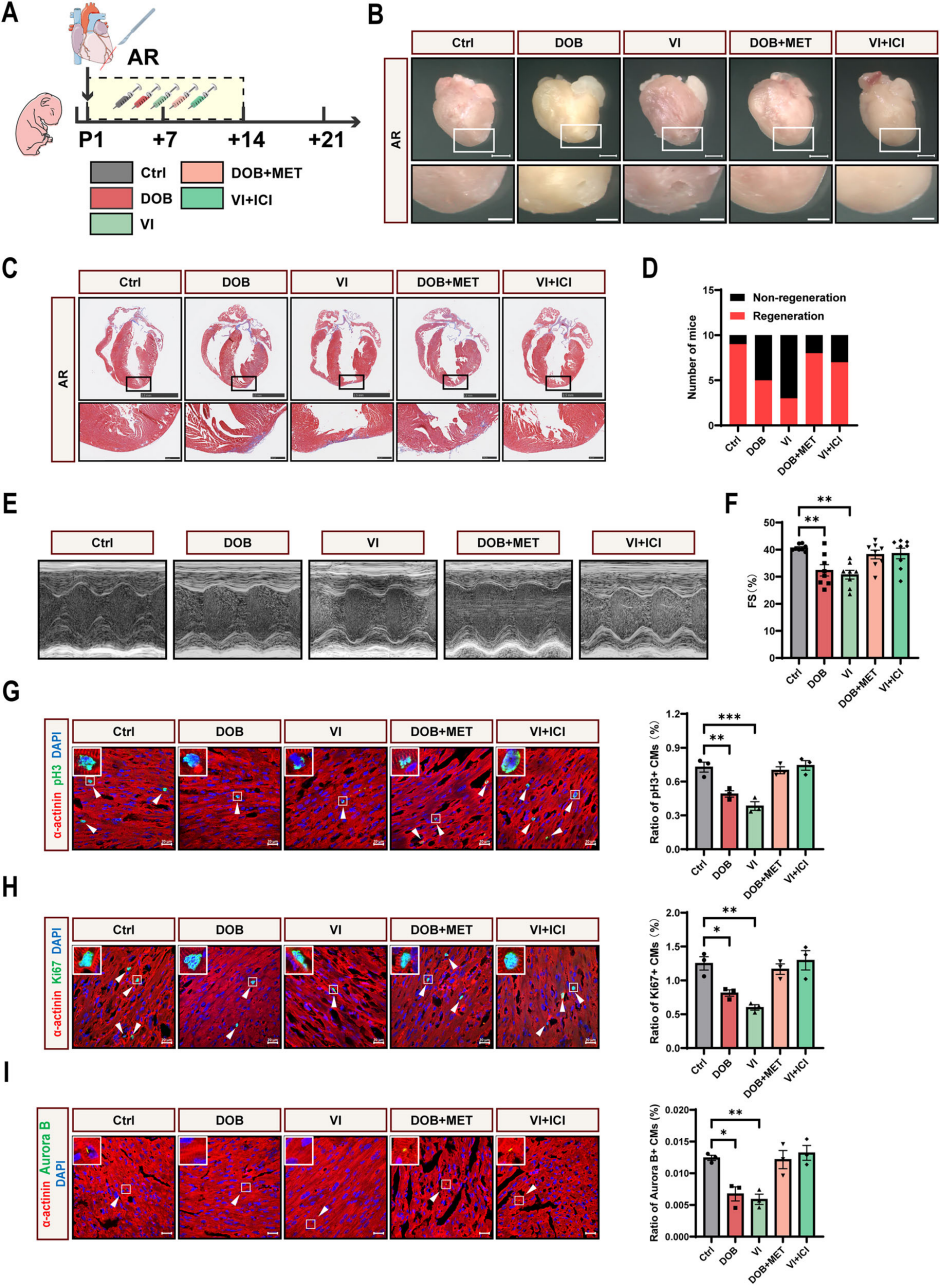
Both β_1_-AR and β_2_-AR inhibit heart regeneration after apical resection. **A** Schematic representation of apical resection (AR) and drug injection for P1 mice. Ctrl, control; DOB, dobutamine, β_1_-AR agonist, 10 mg/kg/d; MET, metoprolol, β_1_-AR antagonist, 10 mg/kg/d; VI, vilanterol, β_2_-AR agonist, 10 mg/kg/d; ICI, ICI-118551, β_2_-AR antagonist, 10 mg/kg/d. **B** Gross morphology of hearts harvested at 21 dpr. Scale bars: upper, 200 μm; below, 100 μm. **C** Masson staining of hearts harvested at 21 dpr. Scale bars: upper, 2.5 mm; below, 250 μm. **D** Statistics of the number of regenerated hearts at 21 dpr (*N* = 10). **E**, **F** Echocardiography analysis of fractional shortening (FS) at 21 dpr (*N* = 8). Immunofluorescence staining of the mitosis marker pH3 (**G**), Ki67 (**H**), and Aurora B (**I**) (green), accompanied with cardiac α-actinin (red) and DAPI (blue) at 7 dpr (*N* = 3). Arrows show cardiomyocytes positive for pH3, Ki67, and Aurora B. CM, cardiomyocyte. Scale bars, 20 μm. Quantitative results were shown as mean ± SEM. Statistical analyses were performed by one-way ANOVA with Tukey’s post-hoc test. *P < 0.05, **P < 0.01, ***P < 0.001.

Next, we further explored the role of different β-AR subtypes (β_1_-AR and β_2_-AR) in heart regeneration. Similarly, P1 mice were subjected to apical resection, followed by β_1_-AR intervention with dobutamine (DOB, β_1_-AR agonist) and metoprolol (MET, β_1_-AR antagonist), and β_2_-AR intervention with vilanterol (VI, β_2_-AR agonist) and ICI-118551 (ICI, β_2_-AR antagonist) (Fig. 3A). Results showed that both DOB and VI inhibited heart regenerative capacity at 21 dpr, and these effects were blocked by MET and ICI, respectively (Fig. 3B–D and S1B). Echocardiography analysis showed reduced systolic function induced by both DOB and VI at 21 dpr, and these effects were also reversed by MET and ICI, respectively (Fig. 3E, F). Further immunofluorescence staining showed that both DOB and VI decreased the proliferation markers (pH3, Ki67, and Aurora B) in heart tissue at 7 dpr, and these effects were counteracted by MET and ICI, respectively (Fig. 3G–I). Notably, VI resulted in poorer heart regenerative capacity, systolic function, and cardiomyocyte proliferation compared with DOB. Collectively, these data demonstrated that both β_1_-AR and β_2_-AR inhibit heart regeneration. β_2_-AR exerts a more potent inhibitory effect on heart regeneration compared with β_1_-AR.

### Both β_1_-AR and β_2_-AR inhibit heart regeneration by reducing YAP expression differentially

**Fig. 4:**
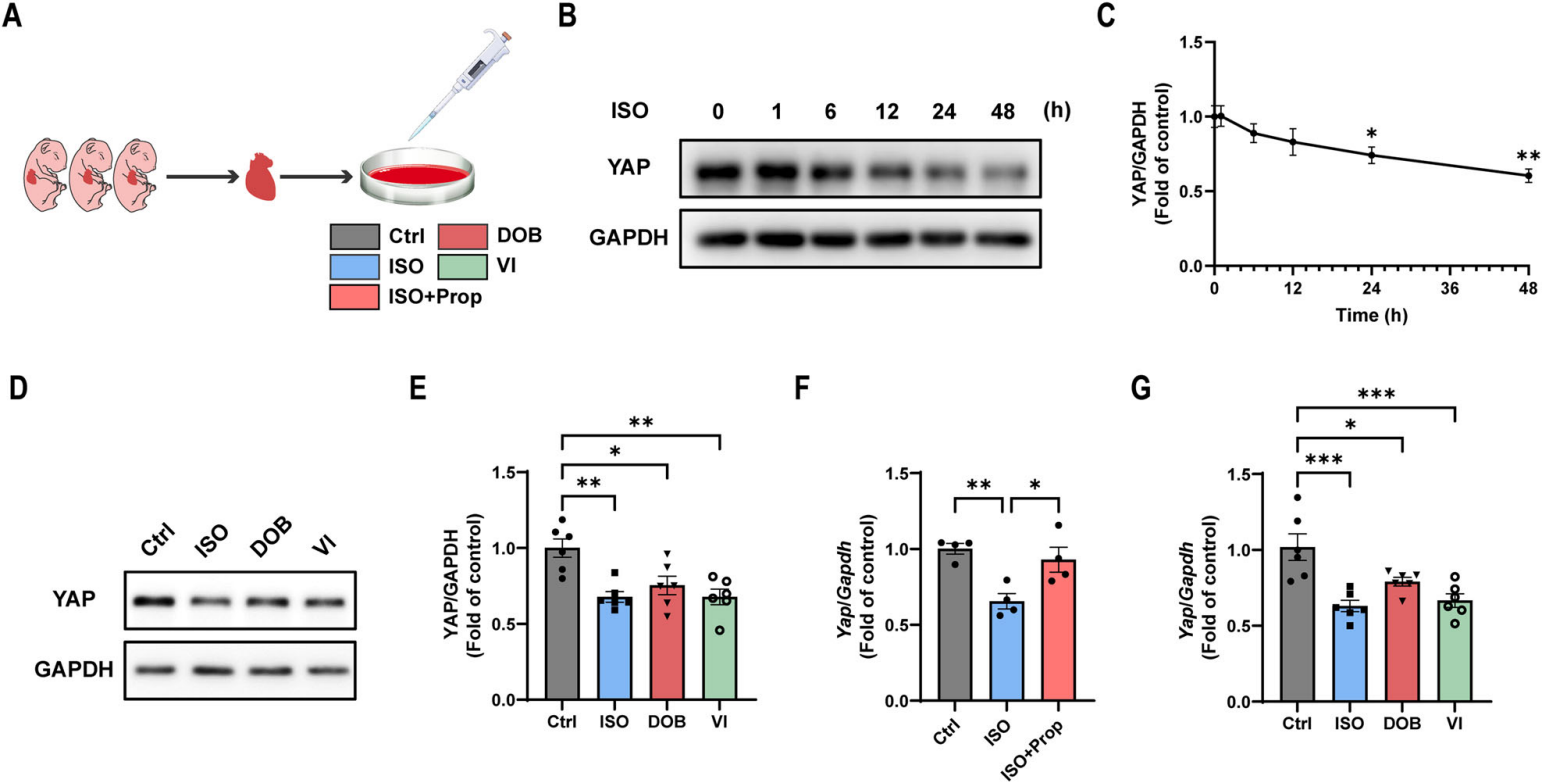
Both β_1_-AR and β_2_-AR reduce the YAP expression differentially. **A** Schematic representation of NMCMs isolation from P1 mice and drug treatment. Ctrl, control; ISO, isoproterenol, β-AR agonist, 10 μM; Prop, propranolol, β-AR antagonist, 10 μM; DOB, dobutamine, β_1_-AR agonist, 10 μM; VI, vilanterol, β_2_-AR agonist, 10 μM. **B**, **C** Western blot analysis of time-dependent YAP expression after ISO treatment (*N* = 5). **D**, **E** Western blot analysis of YAP expression after ISO, DOB, or VI treatment for 48 h (*N* = 6). **F** QPCR analysis of *Yap* mRNA after ISO (and Prop) treatment for 48 h (*N* = 4). **G** QPCR analysis of *Yap* mRNA after ISO, DOB, or VI treatment for 48 h (*N* = 6). Quantitative results were shown as mean ± SEM. Statistical analyses were performed by one-way ANOVA with Tukey’s post-hoc test. *P < 0.05, **P < 0.01, ***P < 0.001.

Next, we explored the underlying mechanism by which β-ARs inhibit heart regeneration. YAP is widely recognized as one of the most critical factors in promoting heart regeneration. To investigate whether β-ARs inhibit heart regeneration by regulating the YAP expression, neonatal mouse cardiomyocytes (NMCMs) were isolated and treated with ISO, and YAP expression was then detected (Fig. 4A). Western blot showed that YAP expression was decreased gradually in a time-dependent manner (0-48 h) with ISO treatment (Fig. 4B, C). Moreover, both DOB and VI reduced YAP expression, with lower YAP expression observed in VI treatment compared with DOB (Fig. 4D, E). In addition, qPCR showed that ISO suppressed *Yap* mRNA level, and the effect was reversed by Prop (Fig. 4F). Moreover, both DOB and VI suppressed *Yap* mRNA level, with lower *Yap* mRNA level examined in VI treatment compared with DOB (Fig. 4G). The above results indicated that both β_1_-AR and β_2_-AR inhibit heart regeneration by reducing YAP expression. β_2_-AR exerts a more potent inhibitory effect on YAP expression compared with β_1_-AR.

### Both β_1_-AR and β_2_-AR reduce YAP expression by downregulating METTL14 and IGF2BP1-mediated *Yap* m6A modification, respectively

We further explored how β-ARs reduce YAP expression. N6-methyladenosine (m6A) modification is the most abundant modification in eukaryotic mRNA, controlling gene expression at the post-transcriptional level [22]. Thus, we reasoned that β-ARs could reduce YAP expression by regulating m6A modification of *Yap* mRNA.

**Fig. 5:**
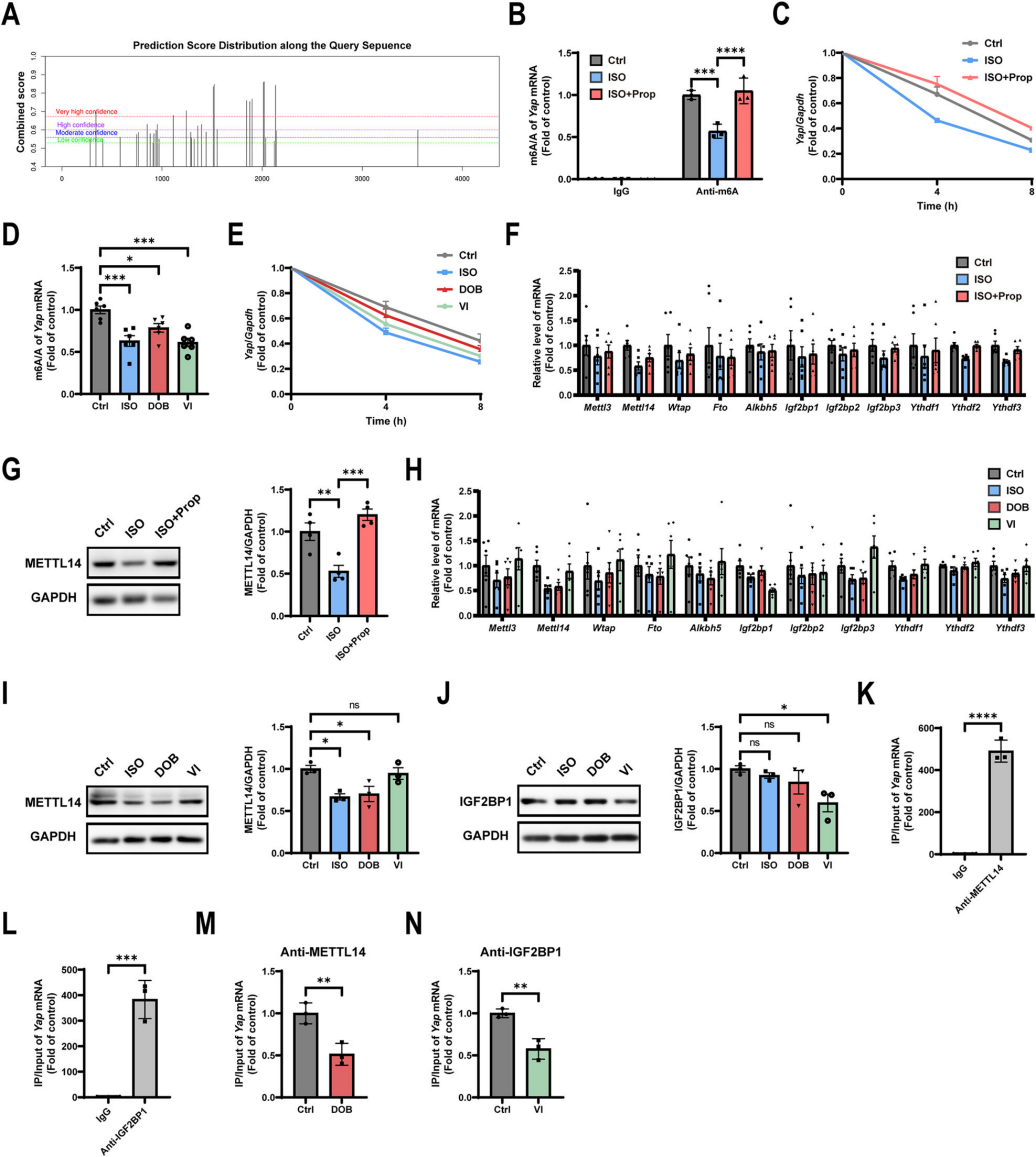
Both β_1_-AR and β_2_-AR reduce YAP expression by downregulating METTL14 and IGF2BP1-mediated *Yap* m6A modification, respectively. **A** Potential m6A sites predicted across *Yap* mRNA according to the SRAMP. **B** MeRIP-qPCR analysis of *Yap* m6A modification after ISO (and Prop) treatment for 48 h (*N* = 3). **C** RNA stability assay of *Yap* mRNA after ISO (and Prop) treatment for 48 h (*N* = 5). **D** MeRIP-qPCR analysis of *Yap* m6A modification after ISO, DOB, or VI treatment for 48 h (*N* = 6). **E** RNA stability assay of *Yap* mRNA after ISO, DOB, or VI treatment for 48 h (*N* = 6). (**F**) QPCR screening of m6A regulators after ISO (and Prop) treatment for 48 h (*N* = 6). **G** Western blot analysis of METTL14 expression after ISO (and Prop) treatment for 48 h (*N* = 4). **H** QPCR screening of m6A regulators after ISO, DOB, or VI treatment for 48 h (*N* = 6). Western blot analysis of METTL14 (**I**) and IGF2BP1(**J**) expression after ISO, DOB, or VI treatment for 48 h (*N* = 3). RIP-qPCR analysis of METTL14 (**K**) and IGF2BP1 (**L**) binding to *Yap* mRNA (*N* = 3). **M** RIP-qPCR analysis of METTL14 binding to *Yap* mRNA after DOB treatment for 48 h (*N* = 3). **N** RIP-qPCR analysis of IGF2BP1 binding to *Yap* mRNA after VI treatment for 48 h (*N* = 3). Ctrl, control; ISO, isoproterenol, β-AR agonist, 10 μM; Prop, propranolol, β-AR antagonist, 10 μM; DOB, dobutamine, β_1_-AR agonist, 10 μM; VI, vilanterol, β_2_-AR agonist, 10 μM. Quantitative results were shown as mean ± SEM. Statistical analyses were performed by one-way ANOVA or two-way ANOVA with Tukey’s post-hoc test. *P < 0.05, **P < 0.01, ***P < 0.001, ****P < 0.0001.

We first analyzed potential m6A sites on *Yap* mRNA sequence using m6A prediction website SRAMP (http:// www.cuilab.cn/sramp) [23]. A total of 34 m6A sites were identified across *Yap* mRNA sequence (Fig. 5A and Table S2). Methylated RNA immunoprecipitation (MeRIP)-qPCR assay confirmed the m6A modification of *Yap* mRNA. ISO significantly downregulated *Yap* m6A modification, and the effect was reversed by Prop (Fig. 5B). Downregulation of m6A modification is known to attenuate mRNA stability and accelerate mRNA degradation [24, 25]. As expected, RNA stability assay showed that ISO promoted the degradation of *Yap* mRNA, and the effect was also blocked by Prop (Fig. 5C). Moreover, both DOB and VI resulted in downregulated m6A modification (Fig. 5D) and stability of *Yap* mRNA (Fig. 5E), with VI inducing a more severe phenotype compared with DOB. These results revealed that both β_1_-AR and β_2_-AR downregulated m6A modification and stability of *Yap* mRNA, thereby leading to reduced YAP expression. β_2_-AR induced the lower *Yap* m6A modification compared with β_1_-AR.

The m6A regulators consist of the methyltransferases (writers), demethylases (erasers), and m6A-binding proteins (readers). As qPCR screened, METTL14, a core subunit of writer complex, exhibited the most significant decrease after ISO treatment (Fig. 5F). Western blot confirmed the reduction of METTL14 after ISO treatment (Fig. 5G). QPCR screening further revealed that, consistent with ISO, DOB also resulted in the most pronounced reduction in METTL14. Notably, in contrast, VI resulted in the most significant reduction in IGF2BP1, one of the readers, rather than in METTL14 (Fig. 5H). Western blot confirmed the decrease in METTL14 after DOB treatment and in IGF2BP1 after VI treatment (Fig. 5I, J). RIP-qPCR analyses further demonstrated the binding of both METTL14 and IGF2BP1 to *Yap* mRNA (Fig. 5K, L). Both METTL14 and IGF2BP1 binding to *Yap* mRNA was weakened after treatment with DOB and VI, respectively (Fig. 5M, N). Thus, these results indicated that β_1_-AR and β_2_-AR specifically downregulate METTL14 and IGF2BP1-mediated *Yap* m6A modification, respectively.

### *Yap* m6A elevation by METTL14 and IGF2BP1 restores YAP expression and cardiomyocyte proliferation inhibited by β_1_-AR and β_2_-AR, respectively

**Fig. 6:**
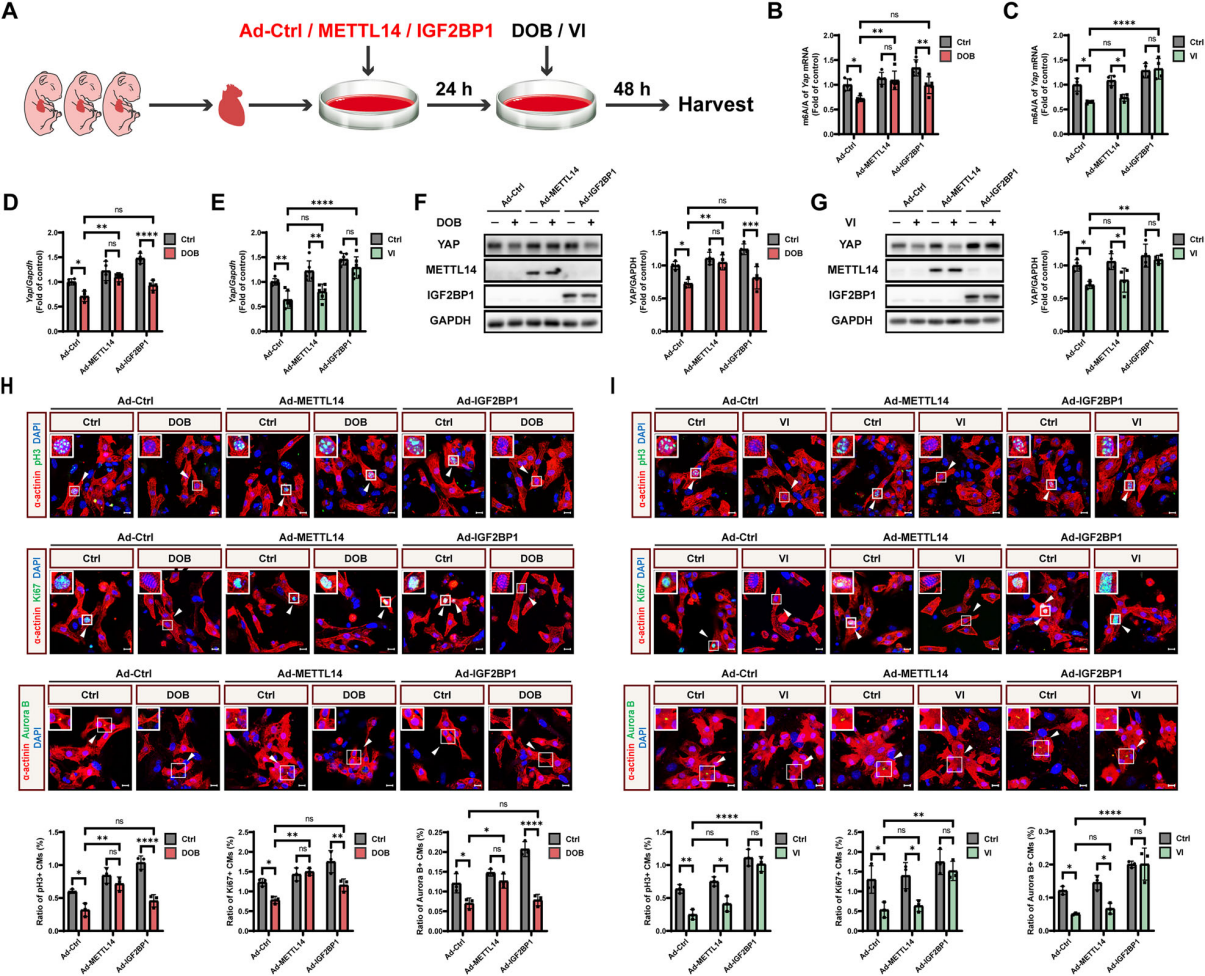
*Yap* m6A elevation by METTL14 and IGF2BP1 rescues YAP expression and cardiomyocyte proliferation inhibited by β_1_-AR and β_2_-AR, respectively. **A** Schematic representation of adenoviruses transfection and drug treatment for NMCMs isolated from P1 mice. Ad adenoviruses, Ctrl control, DOB dobutamine, β_1_-AR agonist, 10 μM; VI vilanterol, β_2_-AR agonist, 10 μM. MeRIP-qPCR analysis of *Yap* m6A modification after METTL14 and IGF2BP1 overexpression and then treated with DOB (**B**) or VI (**C**) for 48 h (*N* = 4). QPCR analysis of *Yap* mRNA after METTL14 and IGF2BP1 overexpression and then treated with DOB (**D**, *N* = 4) or VI (**E**, *N* = 6) for 48 h. Western blot analysis of YAP expression after METTL14 and IGF2BP1 overexpression and then treated with DOB (**F**) or VI (**G**) for 48 h (*N* = 4). Immunofluorescence staining of pH3, Ki67, and Aurora B (green), accompanied with cardiac α-actinin (red) and DAPI (blue) after METTL14 and IGF2BP1 overexpression and then treated with DOB (**H**) or VI (**I**) for 48 h (*N* = 3). Arrows show cardiomyocytes positive for pH3, Ki67, and Aurora B. CM, cardiomyocyte. Scale bars, 10 μm. Quantitative results were shown as mean ± SEM. Statistical analyses were performed by two-way ANOVA with Tukey’s post-hoc test. *P < 0.05, **P < 0.01, ***P < 0.001, ****P < 0.0001.

We next examined whether elevating the *Yap* m6A modification could rescue β_1_-AR or β_2_-AR-inhibited YAP expression and cardiomyocyte proliferation. NMCMs were transfected with adenoviruses encoding METTL14 or IGF2BP1, followed by treatment with DOB or VI (Fig. 6A). Enhanced binding of both METTL14 and IGF2BP1 to *Yap* mRNA was detected after adenovirus transfection (Fig. S3). MeRIP-qPCR showed that METTL14 and IGF2BP1 overexpression elevated *Yap* m6A modification downregulated by DOB and VI, respectively (Fig. 6B, C). Moreover, qPCR showed that METTL14 and IGF2BP1 overexpression increased *Yap* mRNA level reduced by DOB and VI, respectively (Fig. 6D, E). Similarly, western blot showed that METTL14 and IGF2BP1 overexpression enhanced YAP expression decreased by DOB and VI, respectively (Fig. 6F, G). Immunofluorescence staining showed that METTL14 and IGF2BP1 overexpression rescued the proliferation markers (pH3, Ki67, and Aurora B) inhibited by DOB and VI, respectively (Fig. 6H, I). Therefore, these results demonstrated that elevation of *Yap* m6A modification could restore β_1_-AR or β_2_-AR-inhibited YAP expression and cardiomyocyte proliferation.

## Discussion

**Fig. 7:**
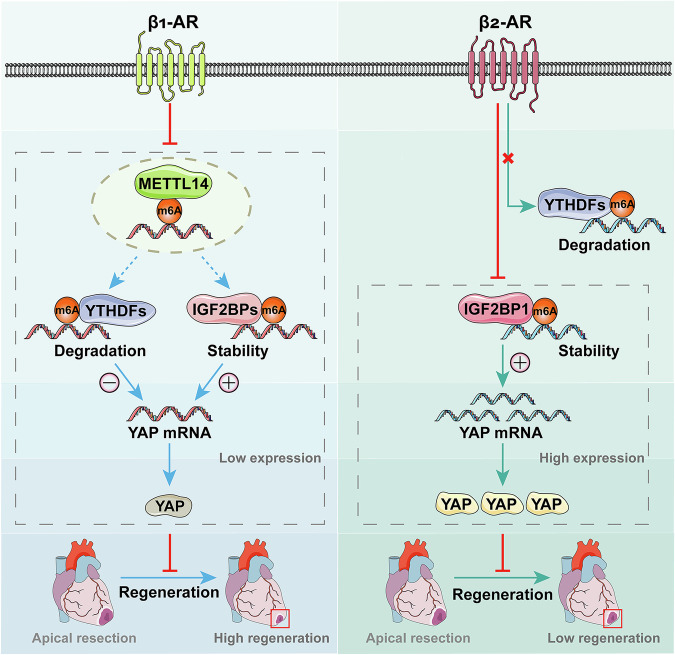
Working model of β_1_-AR and β_2_-AR inhibiting heart regeneration. β_1_-AR and β_2_-AR specifically downregulate METTL14 and IGF2BP1-mediated *Yap* m6A modification, respectively, and then reduce YAP expression and inhibit heart regeneration. Compared with β_1_-AR, β_2_-AR exerts a more potent inhibitory effect on YAP and heart regeneration.

In this study, we revealed that β_1_-AR and β_2_-AR specifically downregulate METTL14 and IGF2BP1-mediated *Yap* m6A modification, respectively, and then attenuate *Yap* mRNA stability, ultimately leading to reduced YAP expression and inhibited heart regeneration. Compared with β_1_-AR, β_2_-AR exerts a more potent inhibitory effect on YAP and heart regeneration (Fig. 7).

Although the regenerative capacity of the adult heart is extremely limited, newborn mammals can completely regenerate the heart after cardiac injury. Exploring the mechanism of heart regeneration in newborn mammals will provide new therapeutic strategy for adult heart injury. In fact, receptors are the most important biomolecules that regulate cellular behaviors. A variety of receptors on cardiomyocytes have been found to regulate cardiomyocyte proliferation and heart regeneration, such as ERBB2 [26], PDGFR-β [27], and OSMR/gp130 [14]. In heart, β-AR is the most critical receptor that mediates the pathophysiological signaling of cardiomyocytes. In this study, we found both β_1_-AR and β_2_-AR inhibit heart regeneration. Consistent with our study, Sakabe et al. [11] reported that β_1_-AR inhibition enhances heart regeneration post myocardial infarction in juvenile mice. Using apical resection model, we confirmed the inhibitory effect of β_1_-AR on heart regeneration. Moreover, we also demonstrated β_2_-AR subtype inhibits heart regeneration, which may account for the difference in β-blockers efficacy of coronary heart disease and heart failure. Currently, although β-blockers have been demonstrated to improve the prognosis of coronary heart disease and heart failure, carvedilol among them is thought to provide better clinical outcomes [28–30]. It may be because carvedilol targets both β_1_-AR and β_2_-AR, potentially promoting heart regeneration more effectively than β-blockers that target only β_1_-AR.

The inhibition of heart regeneration by both β_1_-AR and β_2_-AR is attributed to their reduction of YAP expression. The Hippo pathway is highly conserved to control cell proliferation. As the central effector of Hippo pathway, YAP is widely recognized to promote heart regeneration. Similarly to our study, both β_1_-AR and β_2_-AR are found to activate the Hippo pathway and phosphorylate YAP at S127 [31–34]. The phosphorylation of YAP at S127 could spatially impede the nuclear translocation of YAP, and temporally cause the subsequent ubiquitin-dependent proteolysis of YAP [35]. Besides, we found that prolonged β-AR activation reduces YAP expression, enriching the temporal regulation of YAP by β-AR. The triad suppression of YAP by β-AR may coordinately contribute to the inhibition of heart regeneration. In addition, the effect of β-AR on heart regeneration through YAP suppression should be observed in adult myocardial infarction (MI) model, where sympatho-β-AR activity is enhanced. Although it is known that β-blockers attenuate myocardial damage and long-term pathological remodeling caused by β-AR overactivation to improve cardiac function, it would provide a definitive explanation for the improvement of cardiac function by β-blockers in MI patients from the perspective of heart regeneration.

In addition, we identified m6A modification as a new functional branch downstream of β-AR. In recent years, growing studies have shown that alterations in m6A regulators may affect key signaling factors and mediate disease development [36–38]. We found β-AR could regulate m6A modification of *Yap* by modulating m6A regulators. The establishment of m6A modification as a new functional branch of β-AR enriches the β-AR functional network. Similar to our study, increasing studies show that other receptors, such as melatonin receptor 1 [39], G protein-coupled estrogen receptor [40], and estrogen receptor α [41], could also regulate m6A modification downstream. It suggests that receptor-mediated m6A modification may be a universal pathogenesis and a promising therapeutic target.

Although both β_1_-AR and β_2_-AR inhibit YAP expression and heart regeneration, β_2_-AR exerts a more potent inhibitory effect compared with β_1_-AR. It reflects the functional differences between different β-AR subtypes. Mechanistically, these differences may be ultimately attributed to the distinct m6A regulators affected by β_1_-AR and β_2_-AR, respectively: METTL14 and IGF2BP1. In general, after m6A modification by METTL14, two classes of readers with opposite functions would bind to the m6A site [42]. The binding of YTHDFs leads to degradation of the target mRNA [43], while the binding of IGF2BPs stabilizes the target mRNA [44]. The dynamics of the two readers maintain the balance of mRNA level. It suggests in the context of downregulated m6A modification of *Yap* resulting from METTL14 reduction, YTHDFs and IGF2BPs would still exert mutual constraints and limit the decline of *Yap* mRNA. In contrast, IGF2BPs reduction would directly attenuate the mRNA stability and then result in greatly degradation of *Yap* mRNA. Consequently, the downregulation of *Yap* m6A modification induced by METTL14 reduction would be less severe than that caused by IGF2BP1 reduction, which accounts for the differences in YAP expression and heart regeneration between β_1_-AR and β_2_-AR.

β-AR inhibits heart regeneration in a m6A-dependent manner, which provides a more precise intervention strategy for heart regeneration. The existing β-blockers simply target β-AR and block not only the pathological function but also the normal physiological function mediated by the β-AR. This raises the possibility of side effects from β-blockers and makes severe heart failure contraindicated for β-blockers. Since β-AR inhibits heart regeneration by downregulating *YAP* m6A modification, a better strategy to promote heart regeneration is to restore β-AR-mediated *YAP* m6A modification. A specific β-blocker that could reverse the reduction of m6A regulators by β-AR can be developed for precision medicine.

In conclusion, it is instructive to reveal the mechanism of heart regeneration in newborn mammals for the repair of adult cardiac injury. In this study, both β_1_-AR and β_2_-AR were shown to inhibit heart regeneration in a *YAP* m6A modification-dependent manner. Targeting β-AR-mediated *YAP* m6A modification may become a new strategy for heart regeneration. In the future, more detailed studies will be required to further explore the deep mechanism of β-AR and other receptors regulating m6A modification to find precise drug targets.

## Supplementary information

Supplementary Figures

Table S1

Table S2

Original Western blots

## Data Availability

All data for findings of this article will be shared on reasonable request to the corresponding author.
