# Supplementary material for: β-adrenergic receptor inhibits heart regeneration by downregulating Yap m6A modification
Source: Cell Death Dis. 2025 Apr 14;16(1):294. doi: 10.1038/s41419-025-07642-9 (PMC11997195; doi:10.1038/s41419-025-07642-9)
Supplement: Supplementary file 1 — Supplementary Figures [file 41419_2025_7642_MOESM1_ESM.pdf]

β-adrenergic receptor inhibits heart regeneration by downregulating *Yap* m6A modification

Kaihang Guan[1], Zijian Li[1, 2]*

[1]Department of Cardiology and Institute of Vascular Medicine, Peking University Third Hospital; Beijing Key Laboratory of Cardiovascular Receptors Research; Key Laboratory of Cardiovascular Molecular Biology and Regulatory Peptides, Ministry of Health; State Key Laboratory of Vascular Homeostasis and Remodeling, Peking University; Research Unit of Medical Science Research Management/Basic and Clinical Research of Metabolic Cardiovascular Diseases, Chinese Academy of Medical Sciences, Beijing 100191, China.

[2]Department of Pharmacy, Peking University Third Hospital, Beijing 100191, China.

*Correspondence: lizijian@bjmu.edu.cn

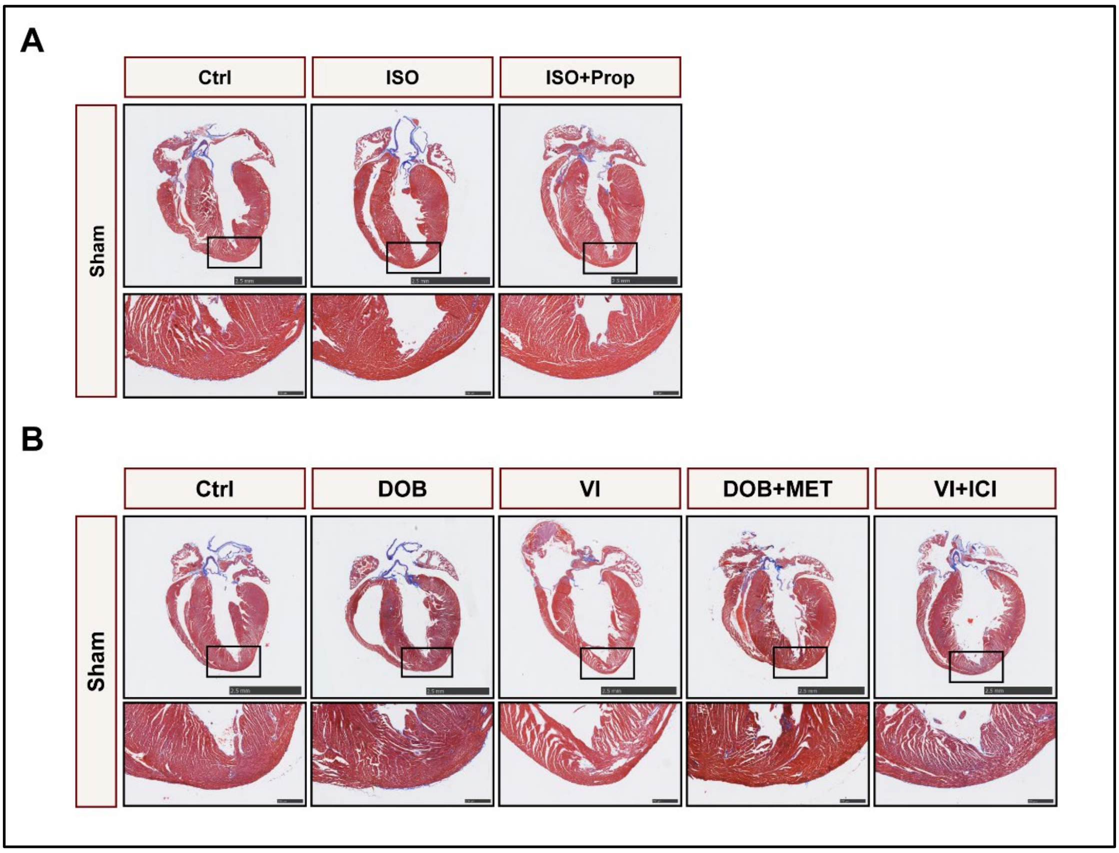

Figure S1. The sham operation of apical resection disease model.

(A) Masson staining of hearts harvested from Ctrl, ISO, and ISO+Prop groups at 21-days post sham operation. Ctrl, control; ISO, isoproterenol, β-AR agonist; Prop, propranolol, β-AR antagonist. Scale bars: upper, 2.5 mm; below, 250 μm. **(B)** Masson staining of hearts harvested from Ctrl, DOB, VI, DOB+MET, and VI+ICI groups at 21-days post sham operation. Ctrl, control; DOB, dobutamine, β_1-AR agonist; MET, metoprolol, β_1-AR antagonist; VI, vilanterol, β_2-AR agonist; ICI, ICI-118551, β_2-AR antagonist. Scale bars: upper, 2.5 mm; below, 250 μm.

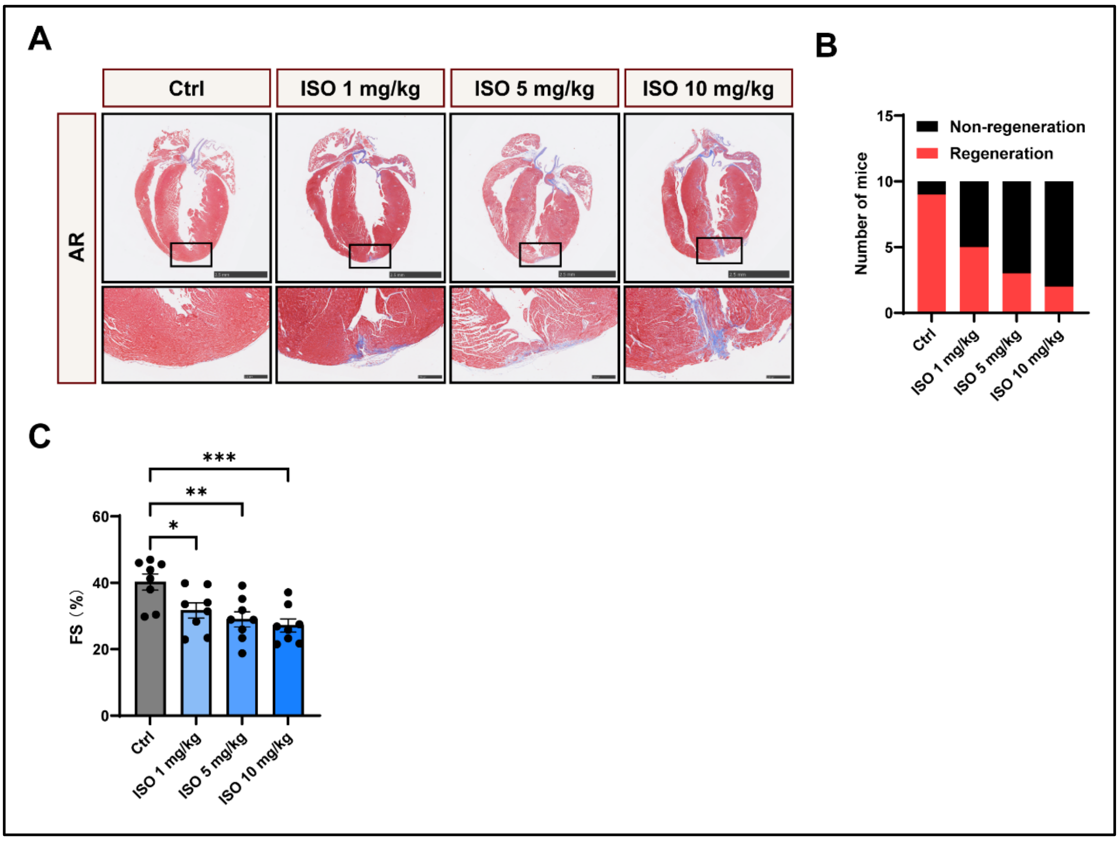

Figure S2. β-AR inhibits heart regeneration upon injection of different ISO doses. (A) Masson staining of hearts harvested at 21 dpr. P1 mice were subjected to apical resection (AR), followed by injection of isoproterenol (ISO) of different doses. Scale bars: upper, 2.5 mm; below, 250 μm. **(B)** Statistics of the number of regenerated hearts at 21 dpr (N=10). **(C)** Echocardiography analysis of fractional shortening (FS) at 21 dpr (N=8). Quantitative results were shown as mean ± SEM. Statistical analyses were performed by one-way ANOVA with Tukey's post-hoc test. *$P<0.05$, **$P<0.01$. ***$P<0.001$.

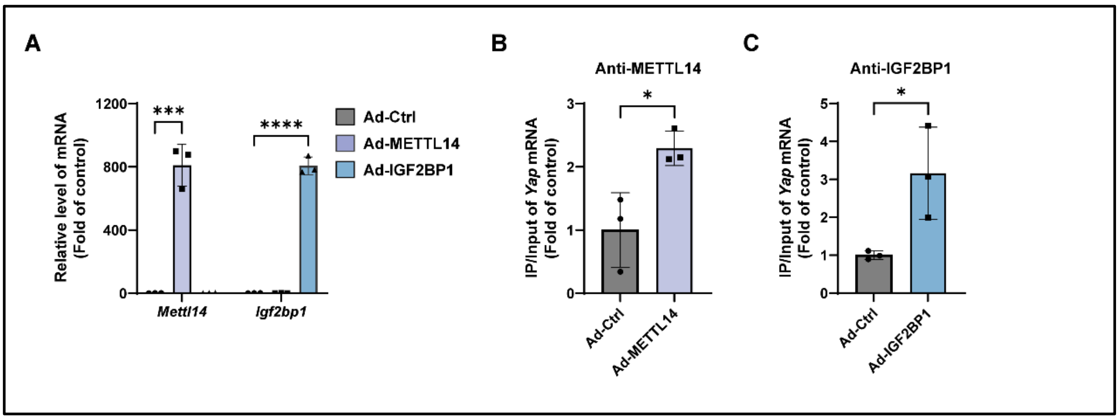

Figure S3. METTL14 and IGF2BP1 binding to *Yap* mRNA was enhanced after overexpression of METTL14 and IGF2BP1, respectively.

(A) QPCR analysis of METTL14 and IGF2BP1 overexpression (N=3). **(B)** RIP-qPCR analysis of METTL14 binding to *Yap* mRNA after transfection of adenoviruses encoding METTL14 (N=3). **(C)** RIP-qPCR analysis of IGF2BP1 binding to *Yap* mRNA after transfection of adenoviruses encoding IGF2BP1 (N=3). Ad, adenoviruses. Ctrl, control. Quantitative results were shown as mean ± SEM. Statistical analyses were performed by two-way ANOVA with Tukey's post-hoc test and two-tailed Student's *t* test. *P<0.05, ***P<0.001, ****P<0.0001.
