# Supplementary figures and images for: β-adrenergic receptor inhibits heart regeneration by downregulating Yap m6A modification
Source: Cell Death Dis. 2025 Apr 14;16(1):294. doi: 10.1038/s41419-025-07642-9 (PMC11997195; doi:10.1038/s41419-025-07642-9)

Figure 4B

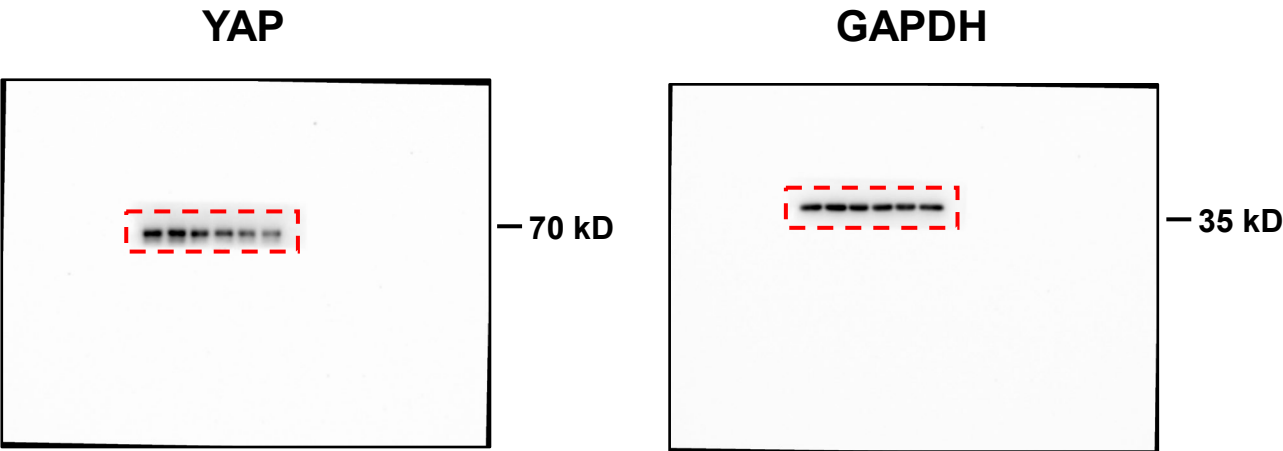

Figure 4D

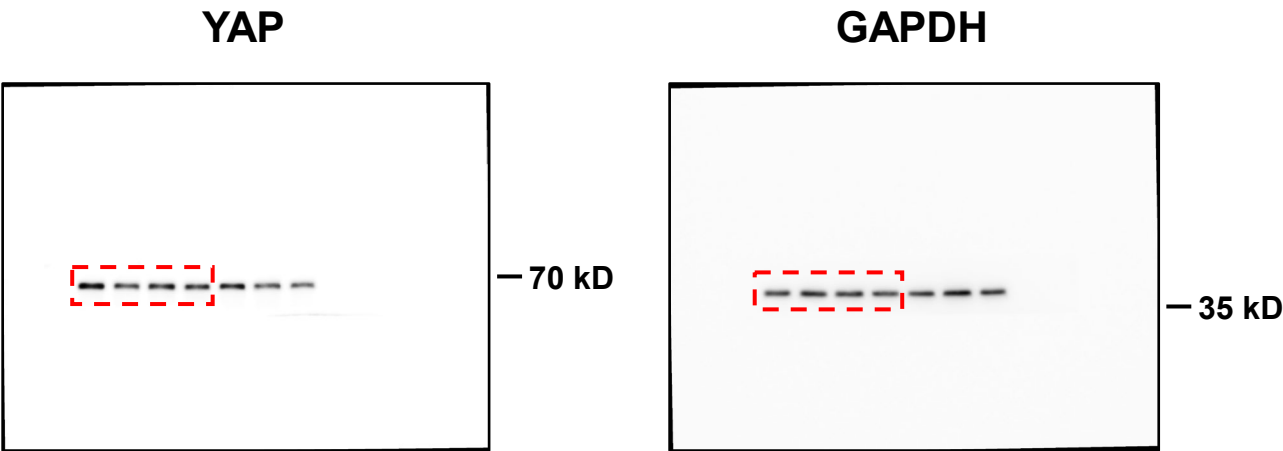

Figure 5G

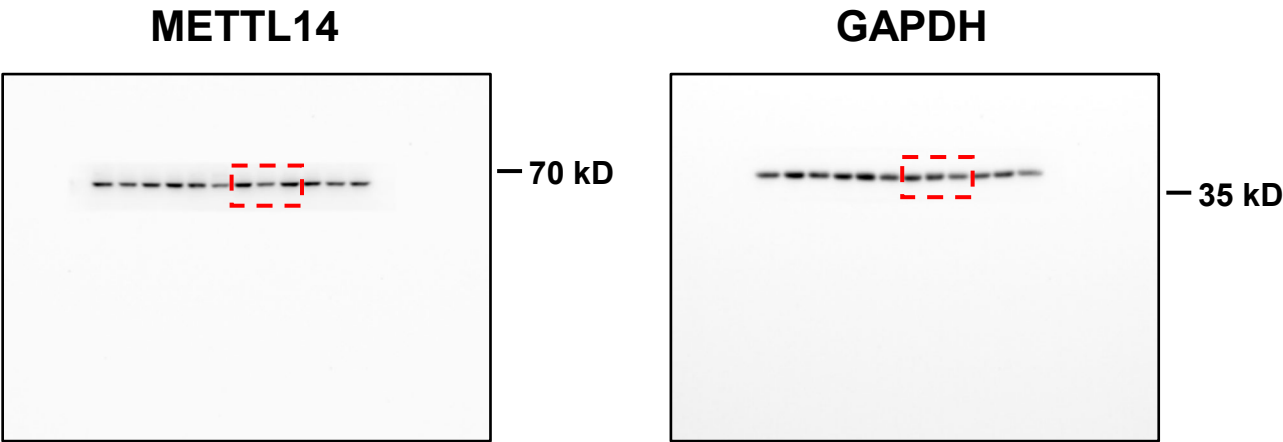

Figure 5J

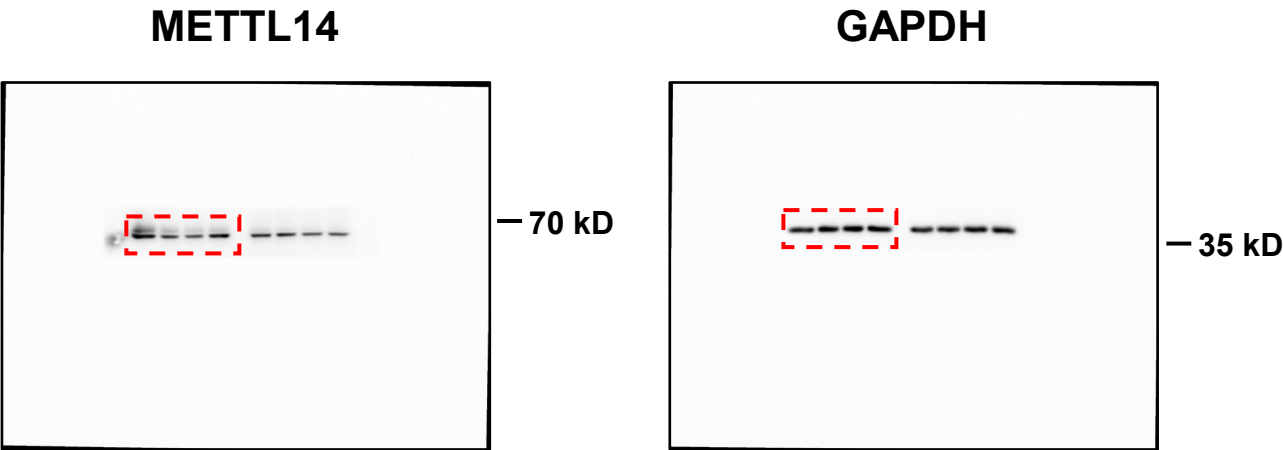

Figure 5K

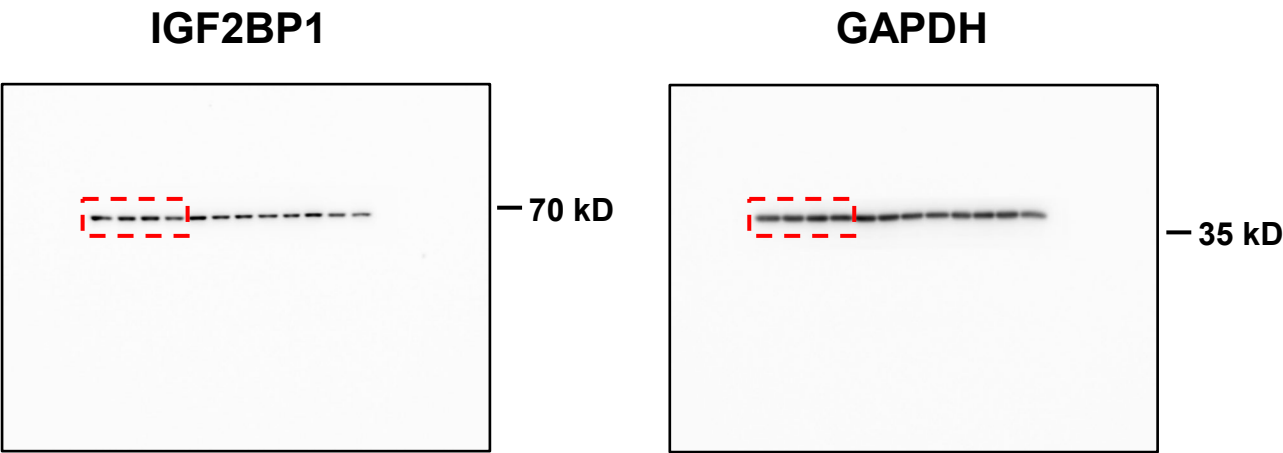

**Figure 6G**

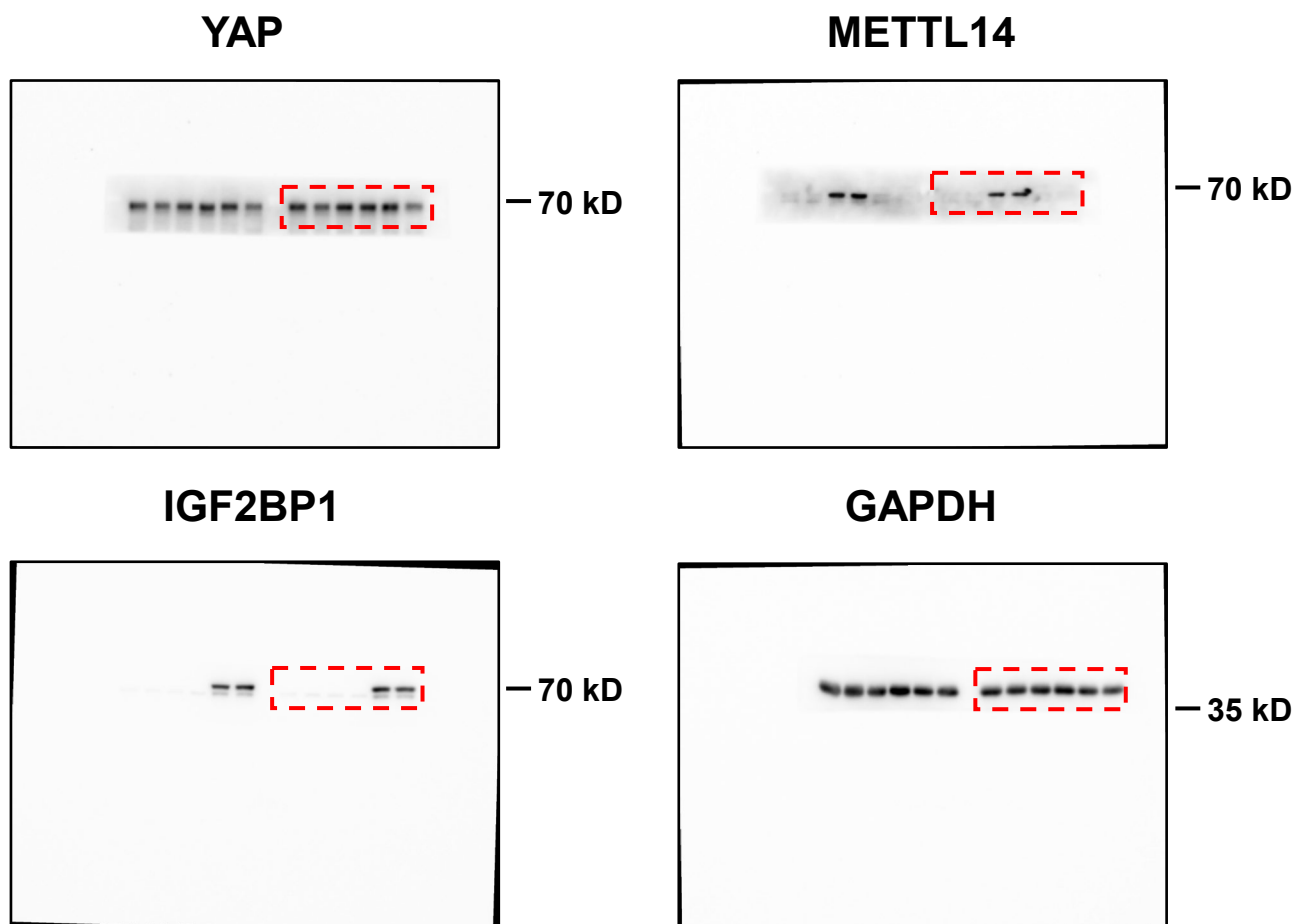

**Figure 6H**

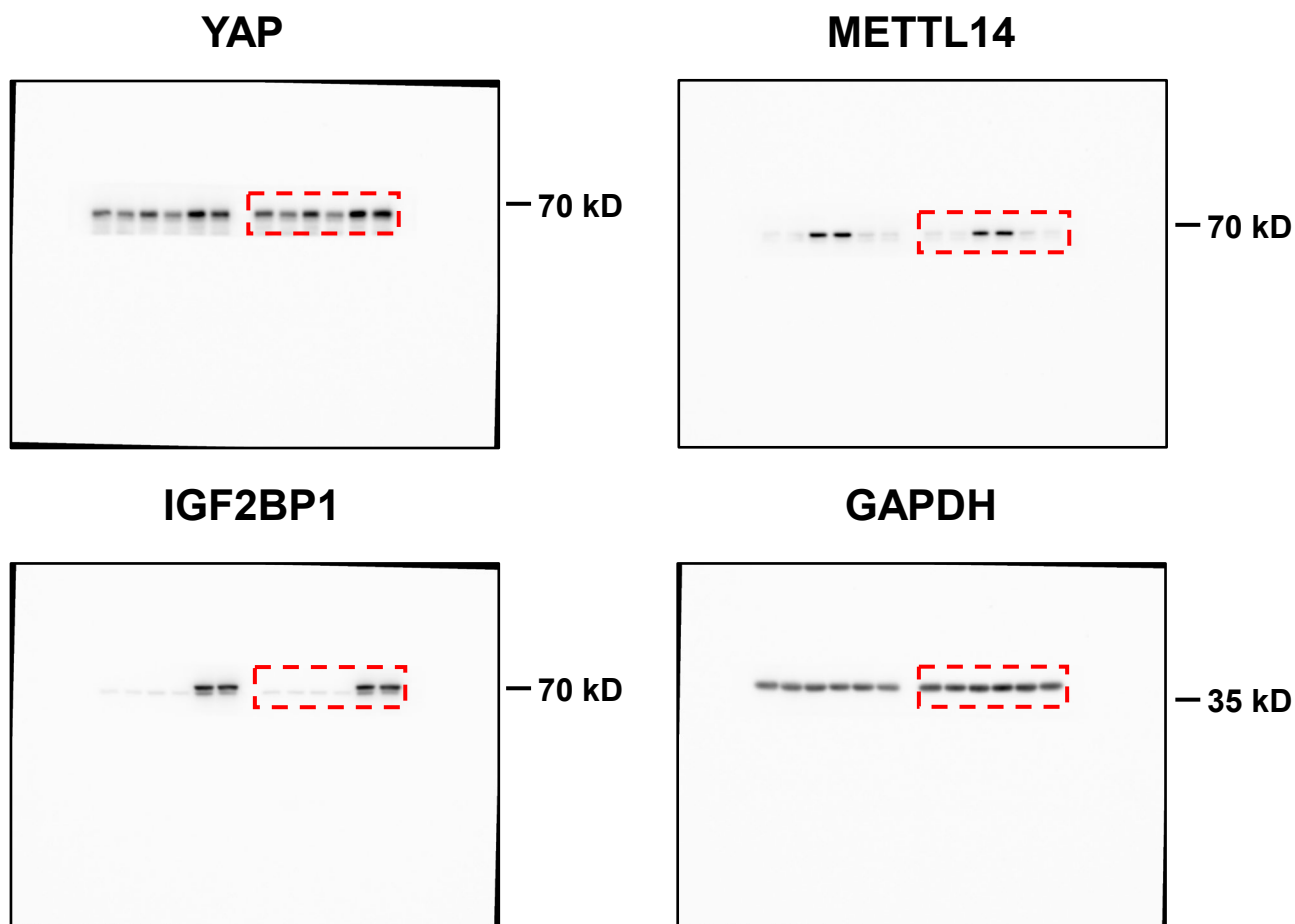

Supplement: Supplementary file 4 — Original Western blots [file 41419_2025_7642_MOESM4_ESM.pdf]
